# Inspecting the Ribozyme Region of Hepatitis Delta Virus Genotype 1: Conservation and Variability

**DOI:** 10.3390/v14020215

**Published:** 2022-01-22

**Authors:** Beatriz Pacin-Ruiz, María Francesca Cortese, David Tabernero, Sara Sopena, Josep Gregori, Selene García-García, Rosario Casillas, Adrián Najarro, Unai Aldama, Adriana Palom, Ariadna Rando-Segura, Anna Galán, Marta Vila, Mar Riveiro-Barciela, Josep Quer, Gloria González-Aseguinolaza, María Buti, Francisco Rodríguez-Frías

**Affiliations:** 1 Liver Pathology Unit, Departments of Biochemistry and Microbiology, Vall d’Hebron University Hospital, 08035 Barcelona, Spain; beatriz.pacin@vhir.org (B.P.-R.); sarasopena91@gmail.com (S.S.); selene.garcia@vhir.org (S.G.-G.); chr.casillas.gmz@gmail.com (R.C.); a.najarro@vhebron.net (A.N.); ualdama@vhebron.net (U.A.); annagalangonzalez@gmail.com (A.G.); marta.vila.salvador@vhir.org (M.V.); frarodri@vhebron.net (F.R.-F.); 2 Centro de Investigación Biomédica en Red de Enfermedades Hepáticas y Digestivas, Instituto de Salud Carlos III, 28029 Madrid, Spain; mmriveir@vhebron.net (M.R.-B.); mbuti@vhebron.net (M.B.); 3 Liver Unit, Liver Disease, Laboratory-Viral Hepatitis, Vall d’Hebron Institut Recerca-Vall d’Hebron University Hospital, 08035 Barcelona, Spain; Josep.gregori@gmail.com (J.G.); josep.quer@vhir.org (J.Q.); 4 Liver Unit, Department of Internal Medicine, Vall d’Hebron University Hospital, 08035 Barcelona, Spain; adrianapalom@gmail.com; 5 Department of Microbiology, Vall d’Hebron University Hospital, 08035 Barcelona, Spain; a.rando@vhebron.net; 6 Center for Applied Medical Research (CIMA), University of Navarra, 31008 Pamplona, Spain; ggasegui@unav.es; 7 Biochemistry and Molecular Biology Department, Universitat Autònoma de Barcelona (UAB), Plaça Cívica, 08193 Bellaterra, Spain

**Keywords:** hepatitis delta virus, ribozyme, next-generation sequencing, quasispecies, conservation, variability, viral fitness, persistence, target, gene silencing

## Abstract

The hepatitis delta virus (HDV) genome has an autocatalytic region called the ribozyme, which is essential for viral replication. The aim of this study was to use next-generation sequencing (NGS) to analyze the ribozyme quasispecies (QS) in order to study its evolution and identify highly conserved regions potentially suitable for a gene-silencing strategy. HDV RNA was extracted from 2 longitudinal samples of chronic HDV patients and the ribozyme (nucleotide, nt 688–771) was analyzed using NGS. QS conservation, variability and genetic distance were analyzed. Mutations were identified by aligning sequences with their specific genotype consensus. The main relevant mutations were tested in vitro. The ribozyme was conserved overall, with a hyper-conserved region between nt 715–745. No difference in QS was observed over time. The most variable region was between nt 739–769. Thirteen mutations were observed, with three showing a higher frequency: T23C, T69C and C64 deletion. This last strongly reduced HDV replication by more than 1 log in vitro. HDV Ribozyme QS was generally highly conserved and was maintained during follow-up. The most conserved portion may be a valuable target for a gene-silencing strategy. The presence of the C64 deletion may strongly impair viral replication, as it is a potential mechanism of viral persistence.

## 1. Introduction

More than 250 million people worldwide are living with the hepatitis B virus (HBV), and between 15 and 20 million of them are chronically co-infected with hepatitis delta virus (HDV). HDV coinfection is associated with a higher risk of cirrhosis, hepatocellular carcinoma (HCC), and liver decompensation, causing the most severe form of viral hepatitis [1]. Like HBV, HDV is differently distributed in the world, with regions with a high prevalence such as Central and West Africa, Central Asia, the Pacific Islands, the Middle East, Eastern Europe, and South America (Amazon basin) [2,3].

HDV is composed of an RNA molecule with high intermolecular self-complementarity, giving rise to a rod-like structure. This 1.2 kb genome presents only one reading frame and codes for a protein existing in two isoforms of different length: the short (S-HDAg) isoform consisting of 195 amino acids (aa) and the long (L-HDAg) delta antigen with 214 aa (27 kDa) [4]. HDV genomic RNA replicates through a rolling circle process mediated by cellular RNA polymerase [5], by producing a concatemer of antigenomic monomers. The individual antigenomic molecules are obtained through a self-cleavage process led by the viral ribozyme [6,7]. Some of these antigenomic monomers are later circularized by a still unclear mechanism [8,9] to be used as templates for the genomic RNA synthesis through another process of rolling circle amplification. The remaining antigenomic molecules enter the transduction process by producing HDAg.

Therefore, an essential step in the viral life cycle is the co-transcriptional self-cleavage activity of the viral ribozyme, which generates both genome and coding viral RNAs. The ribozyme sequence is mainly observed in plant viroids and can catalyze biochemical reactions involved in RNA splicing, gene regulation and other processes [10]. The hepatitis delta virus is the only mammalian virus possessing this auto-catalytic 85-nucleotide-long sequence [10,11]. Notably, its secondary structure is a determining feature for its catalytic activity [12]. It is characterized by four double-stranded domains (P1, P1.1, P2, P3, and P4), three single-stranded regions (J1/2, J1.1/4, and J4/2) and 2 loop regions (L3 and L4) [13]. The ribozyme cleavage site resides in the P1 domain (positions 689/688 and 901/900 in genomic and antigenomic RNA, respectively). The P2 and P4 regions are fundamental for structure stabilization; P3 and J4/2 are involved in catalytic activity, and L3, part of the catalytic site, is essential for the correct RNA split [14,15,16].

Similarly to HBV, HDV shows a high variability and circulates as a population of closely related genetic variants called quasispecies (QS) [17,18]. A mutation rate of 1.4–3.2 × 10^−5^ base substitutions/site/year has been reported for HBV [19], mainly caused by the viral reverse transcriptase which lacks proof-reading capacity. Consequently, a higher QS diversity has been reported in HBV-DNA related to HBV-RNA [20].

The origin of HDV genome variability, on the other hand, is still unclear [21]. Although the cellular RNA polymerase has proofreading activity with a low transcription error rate, HDV shows a high rate of evolution (1.2 × 10^−3^ to 9.5 × 10^−3^ nt substitutions/site/year) [22,23]. As proof of this variability, eight different genotypes have been described worldwide [24], with a divergence of up to 16% within the same genotype and between 20% and 40% between different genotypes [25]. 

The next-generation sequencing technique (NGS) is a very sensitive technique that makes it possible to analyze the less frequent polymorphisms within a variant’s population, providing valuable information on the viral QS and its evolution [26,27,28].

To date, the therapeutic strategy mainly used against HDV infection is based on interferon α, which has a limited administration time due to its adverse effects, without providing long-term suppression of viral replication [29]. Moreover, it has been reported that treatment with nucleotide analogues (NA) targeting HBV reverse transcriptase did not provide an improvement of HBV/HDV patients’ clinical outcome [30]. Notably, new and specific therapeutic options are currently under study or have been recently approved in Europe, such as the Bulevirtide (BLV) (Hepcludex^®^), an acetylated fragment that inhibits viral entry [31]. 

Gene silencing is another promising antiviral strategy [32]. Silencing HDV and HBV expression may inhibit viral expression and limit liver disease progression [21]. However, due to the high viral genome variability, identifying highly conserved regions is essential to designing a strategy that may be effective in the presence of different viral genotypes and quasispecies. Notably, the HDAg region is characterized by high variability, which does not make it an optimal target [33]. On the other hand, due to its essential function in HDV replication, the ribozyme may be a valuable candidate for designing gene-silencing molecules.

We used NGS to analyze the ribozyme region in two longitudinal samples in order to study QS evolution in this region and identify highly conserved regions that may be valuable targets of a gene-silencing strategy.

## 2. Materials and Methods

### 2.1. Patients and Samples

Patients were selected from those attending the outpatient clinics at Vall d’Hebron University Hospital, Barcelona, Spain. Enrolled patients presented at least 3log IU/mL of HDV RNA and presented chronic hepatitis (CHD). Patients with other co-infections or autoimmune disease were excluded. Two plasma samples were collected per patient: one sample at the start of the study and the other at the end, with a mean follow-up of 2.25 years.

### 2.2. Serologic and Molecular Assays

Quantification of HDV-RNA was performed by means of an in-house one-step quantitative RT-PCR technique using the WHO international standard (1st World Health Organization International Standard for Hepatitis D Virus RNA for Nucleic Acid Amplification Techniques-based assays) [34] with linearity ranging from 5.75 × 10^2^ to 5.75 × 10^5^ IU/mL and a detection limit of 5.75 × 10^1^ IU/mL. HBV DNA was quantified using real-time PCR with a detection limit of 10 IU/mL (COBAS 6800, Roche Diagnostics, Rotkreuz, Switzerland). HBV serological markers such as surface antigen (HBsAg) were tested using commercial chemiluminescent assays on a COBAS 8000 analyzer (Roche Diagnostics, Rotkreuz, Switzerland).

The virus was genotyped by analyzing the HDAg region (between nt 910–1270) using next-generation sequencing (Table S1). Genotypes were determined using the Kimura-80 model and a dendrogram was constructed using the unweighted pair group method with arithmetic mean (UPGMA).

### 2.3. Next-Generation Sequencing of the Ribozyme Region

HDV RNA was extracted depending on the sample volume using the automated MagNA Pure LC system (Roche Applied Science, Indianapolis, IN, USA) or by manual extraction following the QIAamp Viral RNA mini kit protocol (QIAGEN^®^, Hilden, Germany). Extracted HDV RNA was denatured at 98 °C for 5 min and immediately transferred to −80 °C and successively retro-transcribed using Accuscript HiFi enzyme (Agilent Technologies, Santa Clara, CA, USA). To amplify the ribozyme (positions 688–771 on the antigenome), three nested PCRs were performed, as shown in Table 1. The first PCR step amplified a larger region (between nt 1454–308) that included the ribozyme portion. In the second amplification, the ribozyme was amplified with the addition of M13 sequences that were used to include the multiplex identifier (MID) specific to each sample in the third and last step. PCR products were charged on 1.5% agarose gel diluted in 1× tris-acetate-EDTA (TAE) buffer (Corning Mediatech Inc., Manassas, VA, USA), analyzed by electrophoresis and purified using the QIAquick Gel Extraction Kit protocol (QIAGEN^®^, Hilden, Germany). Purified samples were fluorometrically quantified with the automated system Freedom EVO^®^ (Tecan, Mannedorf, Switzerland) coupled to the Infinite 200 Pro (Tecan, Mannedorf, Switzerland) fluorimeter using the Quant-iTTM PicoGreen^®^ dsDNA Assay Kit (Thermo Fisher Scientific, Camarillo, CA, USA).

Samples were then normalized to the concentration of 1.00 × 10^10^ molecules/μL. Samples were pooled and then processed using NGS according to the Miseq illumina platform protocol (Illumina, San Diego, CA, USA).

### 2.4. Quasispecies Analysis and Statistics

The sequences (reads) obtained were bioinformatically filtered (R software [35]). Only those reads with a complete sequence, a good overlapping between forward and reverse strands (less than 10% of mismatches), and optimal quality were maintained and demultiplexed to obtain the unique QS sequences (haplotypes).

QS conservation was analyzed by calculating the information content (IC) of each position in a multiple alignment of all haplotypes obtained by means of NGS, followed by a sliding window analysis, as previously described by our group [36].

Inter-patient and intra-patient sequence distance was studied to evaluate QS variability. Moreover, different complexity indices were also considered, as previously reported by our group [37]: number of reads per sample, number of master reads (Mstr), master percentage (Mpct), number of haplotypes, polymorphic sites, number of mutations, Shannon index, Gini–Simpson coefficient, functional attribute diversity (FAD), mutation frequency (Mf), nucleotide diversity (Pi) and Pi to Mf ratio. The mutations in ribozyme sequences were identified by aligning the QS sequence for each sample and each patient with its specific genotype consensus. Mutation frequency was obtained by summing the relative frequencies of each haplotype carrying the specific mutation.

### 2.5. In Vitro Test of Mutations

To evaluate the effect of the observed mutations in HDV replication capacity, the most relevant changes were introduced using site-directed mutagenesis (QuickChange Lightning site-directed mutagenesis kit -Agilent Technologies, Waldbronn, Germany), in a plasmid (pCMV-HDV-1.2X) containing 1.2 copies of genomic cDNA [38], following the manufacturer’s protocol. The correct introduction of the mutation was ensured by analyzing the plasmid using Sanger sequencing. Mutated plasmids were extracted using the Endotoxin free NucleoBond Xtra Midi Plus kit (Machery-Nagel, Düren, Germany). The concentration (ng/μL) of the extracted plasmids was determined by fluorometric quantification using Qubit fluorometers (Thermo Fisher Scientific-Life Technologies, Waltham, MA, USA).

Hepatocarcinoma Huh7 cells were cultured with Dulbecco Eagle’s minimal essential medium (DMEM) supplemented by 10% of fetal bovine serum (FBS) and penicillin (100 U/mL), streptomycin (100 μg/mL), and Glutamax (2 mM). Cells were plated at 160,000 cells/mL and transfected with wild-type (wt) and mutated plasmids using the Magnetofectamine O2 kit (OZbiosciences, Marseille, France) according to the manufacturer’s protocol. To guarantee production of HDV viral particles, a plasmid containing 1.3-length HBV genome (pTriEx-HBV) [39] was included in each condition. The supernatant was collected 72 h after transfection.

To quantify HDV RNA release in vitro, viral RNA was extracted from the cell supernatant using the automated MagNA Pure LC system (Roche Applied Science, Indianapolis, IN, USA). To ensure plasmid removal, a DNAse step of the extracted RNA was performed (DNAse I, Amplification grade, Thermo Fisher Scientific, Camarillo, CA, USA). HDV RNA was then quantified as reported above for plasma samples. To ensure that transfection did not affect cell viability, control negative cells were transfected with the empty pCMV backbone.

## 3. Results

### 3.1. Patients and Sequencing

Twenty-five patients were included in the study with a total of 50 samples. Most patients were infected by genotype 1, but 1 patient presented genotype 8 HDV (P04). However, after applying the quality filters, only 19 patients (38 samples) were later considered. Notably, all of them were infected by genotype 1 virus. HDV viremia did not change between the two timepoints (median [IQR] log10 HDV RNA of 5.76 (5.02–5.83) and 5.76 (3.68–5.76), respectively (Table 2).

Among the 38 samples that passed this filter, we obtained a median (IQR) of reads of 4550.5 (1342.24–5892.74) per patient.

### 3.2. Ribozyme Conservation

**Figure 1:**
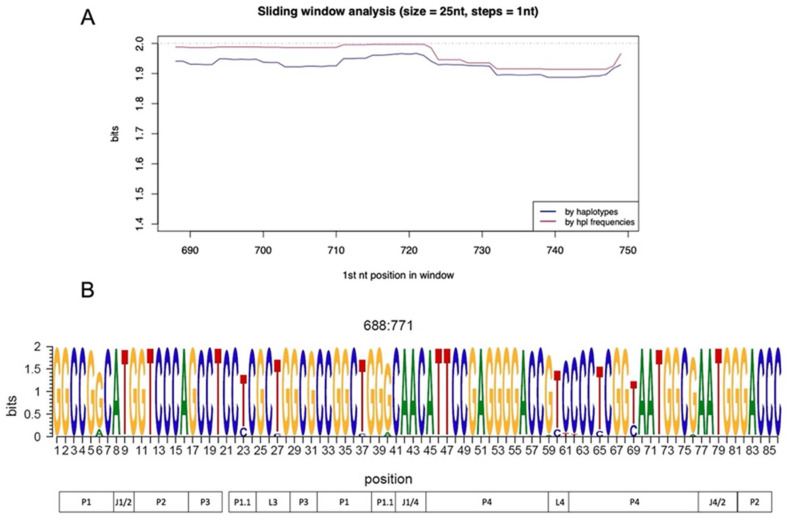
Conservation of ribozyme region by sliding window analysis and represented by logo. (**A**) The sliding window analysis is the result of the mean information content (bits) of the 25-nt windows with a displacement between them of 1-nt obtained by multiple alignments of all the quasispecies (QS) haplotypes. The analysis was implemented by considering (red line) or not (blue line) haplotypes frequency. The dashed line represents the maximum level of conservation (2 bits). (**B**) Logo representation of the nucleotide sequence corresponding to the entire ribozyme region from the genome positions 688 (corresponding to position 1 in the ribozyme) to 771 (corresponding to position 86 in the logo representation). The height of each letter represents the grade of conservation from a maximum of 2 bits to a minimum of 0. The sequence is shown in the genome sense. The different structural and functional domains of the ribozyme are reported at the bottom [40].

QS conservation was analyzed by aligning all the haplotypes, applying a sliding window analysis, and calculating the information content considering their relative frequency or not. The ribozyme (between positions 688 and 771) was overall highly conserved and 85% of the nucleotide positions presented 2 bits of information content (100% conservation) (Figure 1A). No difference was observed between considering or not considering haplotype frequency. Of the 85 nucleotides included in the ribozyme sequence, just 3 nt positions (around 3.5% of the total) presented a conservation of less than 1.5 bits (Figure 1B).

**Figure 2:**
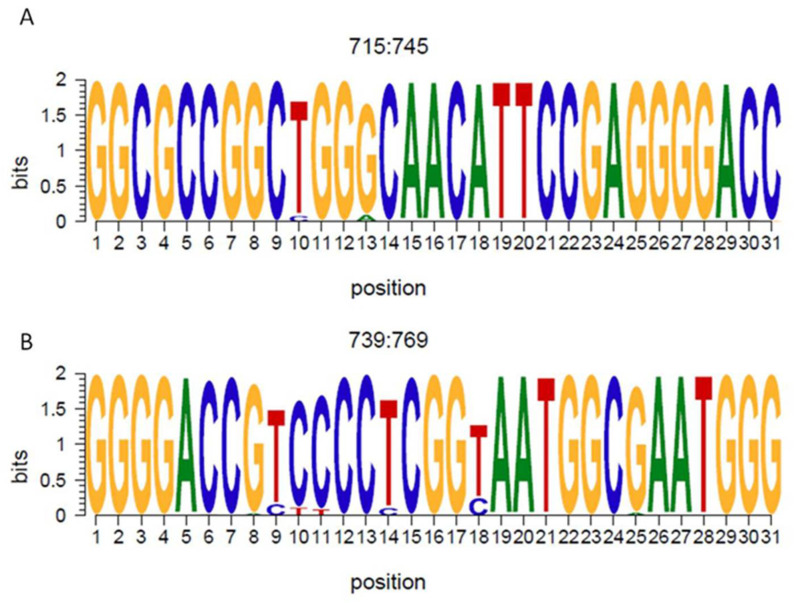
Logo representation of the most conserved and most variable portion of the ribozyme. The logo represents the most conserved (**A**) (nt 715–745) and most variable (**B**) (nt 739–769) regions of the ribozyme. The height of each nucleotide represents its information content in bits (2 bits indicates 100% of conservation). The sequence is shown in the genome sense.

The most conserved region was between nt 715–745 (Figure 2A), where 100% of the nucleotide presented a conservation of more than 1.5 bits. The most variable region encompassed positions 739 and 769, as described previously [41]. In this region, 3 nt positions presented a conservation of less than 1.5 bits (3/31 nts), although 80% (25/31) of the nts showed a high level of conservation (Figure 2B).

### 3.3. Ribozyme Quasispecies (QS) Evolution and Variability during Follow-Up

**Figure 3:**
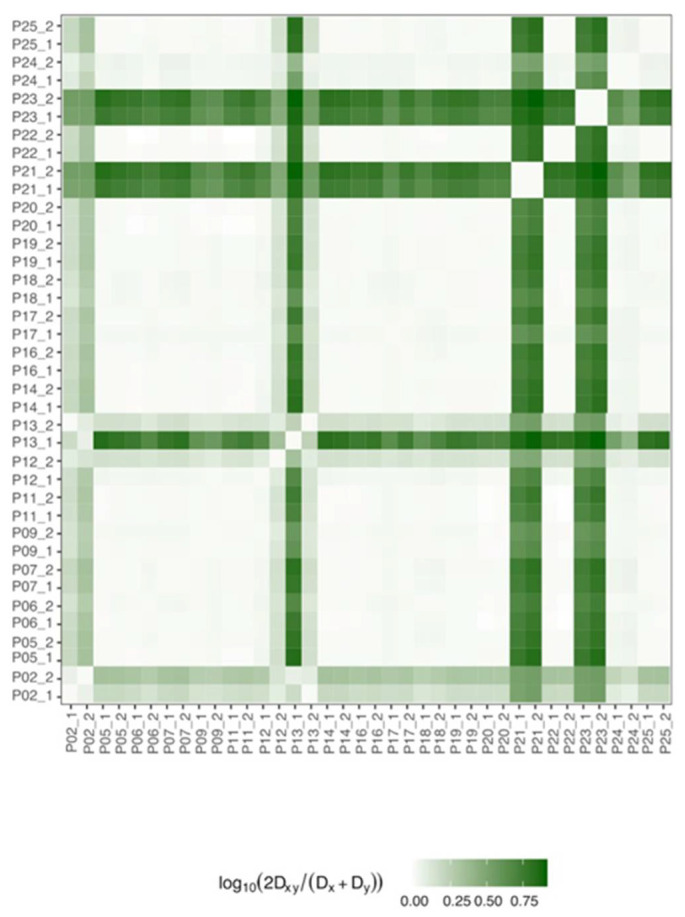
Sequences distance between samples. The heatmap shows the distance of the ribozyme sequences between the different samples included in the study as the logarithm of the ratio between QS nucleotide distance (D_xy_) and the nucleotide diversity average of each quasispecies (D_x_ and D_y_). The tone of the green indicates the degree of distance, from the lighter green, which corresponds to a shorter distance, to the darker green, which indicates greater sample-sample distances. Distance was calculated by considering the QS consensus of each sample.

The genetic distance between samples was determined to evaluate ribozyme QS evolution during the patient’s follow-up (Figure 3).

As expected, considering the high level of conservation, no distance (<0.25) was observed between the 2 samples for each patient (Figure 3). Although patients 2, 12, 13, 21, and 23 presented a greater distance (>0.50) compared to the others, when their sequences were examined in greater depth, this greater distance was determined by just a few nucleotides.

To inspect even more ribozyme QS variability, different complexity indices were analyzed and compared between sample A and sample B. Notably, no difference was detected for any tested index (Table S2).

### 3.4. Analysis of Mutations

Mutations were identified by aligning haplotypes sequences with the corresponding genotype consensus. Although the ribozyme was conserved overall, when studying each sample individually, a total of 48 mutations were observed. Of these, 12 were in at least six patients (Table 3). When looking at mutation type, we observed that the most observed changes were transitions: C→T (16 mutated positions), G→A (10 mutated positions) and T→C (9 mutated positions) (Figure S1). Moreover, four of the 12 mutations observed involved the P4 domain, as reported in previous studies [42].

**Figure 4:**
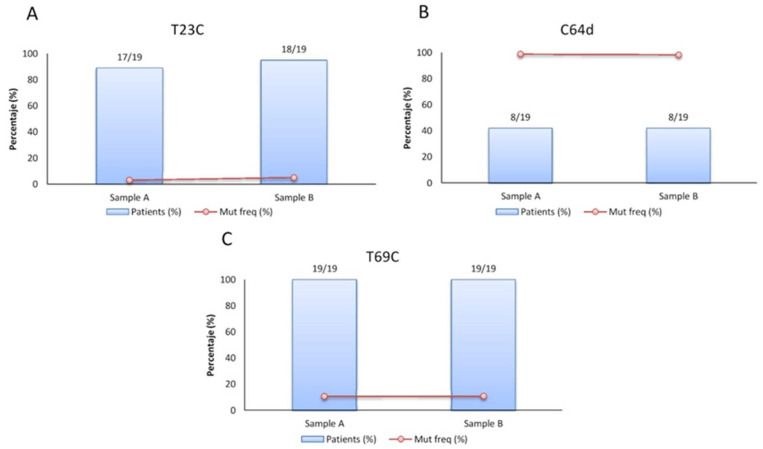
Incidence of the principal mutations observed in the ribozyme. Graphs show the relative frequency (red line), the percentage (%) and number of patients (blue bar) presenting the mutation T23C (**A**), C64d (**B**) and T69C (**C**) and how they change between the 2 time points (between sample A and sample B).

Of the observed mutations, only three involved at least eight patients and were maintained or selected (mutation frequency that changes between the two-time point) in the follow-up samples: T710C (position 23 if considering the first nt in ribozyme starting from 688), T756C (T69C) and the deletion in position 751 (C64d). The T23C mutation involved 17 of the 19 patients in sample A with a mean frequency of 2.99%, and 18/19 of the patients with a mean frequency of 4.96% in sample B (Figure 4A). The T69C mutation is found in both samples in 100% of patients, with a relative frequency that was maintained between the two samples in the follow-up (10.6% and 10.8% in sample A and B, respectively) (Figure 4C). The deletion in position 64 was observed in eight of the 19 patients in both samples with a frequency of 98.7% in sample A and 98.3% in sample B (Figure 4B).

Although the relative frequency of mutations is generally maintained, in some patients some mutations were positively or negatively selected, such as the T23C mutation in patient 2 (from 5.29% to 17.07%), and in patient 12 (from 9.52% to 36.97) (Figure S2A) and the T69C mutation in patients 2 (from 43.88% to 71.85%) and patient 12 (from 2.79% to 24.11%) retracted in patient 13 (from 91.15% to 39.09%) (Figure S2C).

### 3.5. In Vitro Test of Mutations

**Figure 5:**
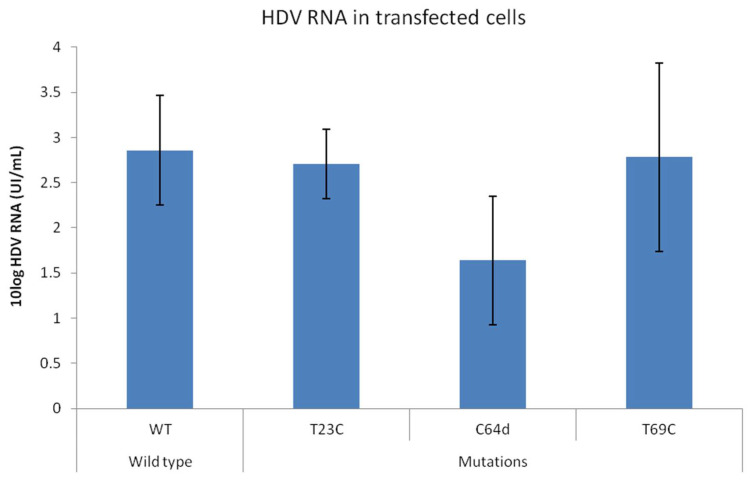
Effect of the most relevant observed mutations in HDV replication in vitro. The graph shows HDV titer in cell supernatant after transfection with HBV and HDV wt or mutated *p* values were obtained by applying the Mann-Whitney test.

Considering the high degree of ribozyme conservation, the observed mutations may potentially affect HDV fitness, thus promoting or inhibiting viral expression. To test the effect of the three detected principal mutations (T23C, C64d, T69C) of HDV expression, the plasmids carrying the desired mutations will be transfected in the presence of HBV and the HDV RNA titer was quantified in transfected cell supernatants 72 h after transfection. We efficiently expressed HDV in vitro, obtaining a mean ± SD titer of 2.86 ± 0.61 IU/mL 72h after transfection. The T69C mutant showed a similar replication rate than wt (2.78 ± 1.04 UI/mL, *p* = 0.662). Of note, in the presence of the mutation T23C (2.7 ± 0.38 IU/mL, *p* = 1), the HDV titer was reduced around 1.05-fold. The interference with viral expression was even more strong in presence of the deletion in position 64 (C64d) that caused a reduction in viral expression of around 1.22log (HDV RNA = 1.63 ± 0.71 UI/mL, *p* = 0.08) (Figure 5).

## 4. Discussion

Due to the lack of a viral polymerase, no specific antiviral therapy is available against HDV infection. New treatment options against HDV are currently under study, such as HBsAg release, prenylation and viral entry inhibitors [43]. Among them, the entry inhibitor BLV has been recently approved for use, alone or in association with peg-IFNα, in CHD patients with compensated liver disease [44,45]. 

Notably, a gene therapy approach may be a valuable strategy to promote HDV RNA elimination and block disease progression. Due to their intranuclear location, the genome and antigenome seem to be resistant to interfering RNA (siRNA) activity [46], whose silencing activity is developed in the cell cytoplasm. HDV genomes, however, may be targeted by antisense oligonucleotides (ASOs), which are active within the nucleus [47]. Although inhibition of the expression of HBsAg through a siRNA may interfere with HDV infection [48], a combination of silencing molecules targeting both viruses may represent a highly valuable therapeutic strategy. To date, however, no silencing HDV-specific molecules have been reported. The extreme variability of HDV RNA may make it very difficult to design an effective antisense oligonucleotide. To this end, identifying highly conserved regions in the HDV genome to use as target may be essential.

Ribozyme activity is crucial to producing the excision of unitary RNA monomers during viral replication. Given its key role, this region may be a valuable target for antisense oligonucleotides. Although the HDV genome has been extensively studied using Sanger sequencing [42,49], this is the first report to focus on studying ribozyme QS conservation and variability using next-generation sequencing. As expected, the ribozyme was overall highly conserved, with a hyper-conserved region encompassing positions 715–745. This region involves the P1 domain, which is essential for ribozyme activity since it is the region where the self-cleavage site resides [50].

To evaluate ribozyme QS evolution during follow-up, two samples were included per patient. Notably, when considering sequence distance or complexity indices, no differences were observed between the two longitudinal samples, suggesting that the QS in this genome portion did not vary over time.

However, a variable region between nt 739–769 was also identified, although changes were observed in just a few nucleotide positions (specifically in six positions). This variable region falls into the P4 structural domain as observed in a previous study that reported a higher mutations level for both genomic and antigenomic RNA [42]. Although this domain is not directly involved in the self-cleavage process, it has been reported that the conservation of the nucleotide sequence is essential for a stable base pairing, which is a necessary condition for efficient self-cleavage activity [51].

A recently reported study shows that a wt ribozyme sequence guarantees the optimal ribozyme activity in co-transcriptional conditions [52]. Different mutations were experimentally introduced in ribozyme sequence to study its functional domains, demonstrating that some positions cover important roles in cleavage activity such as the U23 and C24 in the L3 domain [53] or the T20, C21 and C75 which are involved in coordinating Mg^2+^ ion and cleavage catalysis [54]. By analyzing the HDV full genome in CHD patients, different mutations were found in the ribozyme sequence [42]. Notably, many of these identified mutations were also observed in this study. We identified a total of 48 mutations, 12 of which were found in at least six patients. The identification of mutations in such a conserved and active region of the HDV genome may indicate that they do not affect viral fitness. As previously reported [42], the observed mutations mainly involved the P4 domain (positions 60, 61, 62, 64, 65 and 69). Of these 12 mutations, three presented a relative frequency of 1% or more and were tested in vitro: T23C (in L3 domain), C64d, and T69C (both in P4 domain).

In vitro testing of the ribozyme self-cleavage activity in the presence of the T23C mutations showed a decrease of 10^3^-fold of the cleavage rate [53], however when we tested the viral replication in vitro in the presence of this mutation, we observed that the change in position 23 had a limited impact on viral replication. This mutation falls into the L3 domain, which is an important element in the cleavage activity [16,48]. Of note, as previously reported, this change is less effective in interfering with ribozyme activity than others (in positions 20, 21, and 25) that may entail the loss of ribozyme activity [42].

Changes in positions 64 and 69, however, involved the P4 domain. Higher mutation levels in this domain have been previously reported [42]. As previously mentioned, this domain shows a stabilizing function of the ribozyme structure, and it is not directly involved in RNA catalysis. Indeed, it has been observed that its elimination does not compromise virus replication [15,16]. Although the presence of the T69C change did not impact viral replication, the deletion in position 64 strongly reduced HDV replication by more than 1log IU/mL. Considering the non-essentiality of the P4 domain, the presence of this 1nt-long deletion in this portion could probably alter the ribozyme secondary structure, thus affecting viral replication. Moreover, this change was observed at a relative frequency of around 50%. The fact that it had been observed and maintained at such a high frequency in viral QS suggests that this mutation may promote viral persistence, as has been reported for other viruses such as hepatitis C [55] and human immunodeficiency viruses [56].

This is the first study to focus on studying ribozyme QS using NGS, but it does have some limitations. Although 25 patients were selected, only 19 of them were correctly amplified and passed the quality filters. In addition, all patients were infected with HDV genotype 1, which is the main genotype in infected patients in Spain [57]. More patients, infected with more viral genotypes should be included to confirm these results. Moreover, in silico modeling should be implemented to highlight the effects of the C64 deletion on the secondary structure and, consequently, on the function of the ribozyme.

In conclusion, as expected considering its essential role in the viral life cycle, the ribozyme is overall highly conserved in viral QS and did not change over time. The most conserved portion involved a domain that plays a direct role in the auto-catalysis process and may be a valuable target for designing a new gene-silencing strategy against HDV. The most variable region, however, involves a domain that is not essential from a functional point of view, but a deletion in this region may strongly impair viral replication. Considering its relatively high frequency, it may be a potential mechanism of viral persistence.

## Figures and Tables

**Table 1:** Protocols for ribozyme amplification. The table shows the different steps for ribozyme amplification, including the retro-transcription and the three nested-PCR (1st PCR; M13 PCR, MID PCR). The primer sequence and amplification region for each step is shown. The M13 sequence tail is underlined. Abbreviations: Fw indicates forward; Rv, reverse; MID, multiplex identifier.

Amplification Step	Primer	Amplified Region	Primer Sequence (5′→3′)	Protocol
RT	RT rv	1435–1454	TGGCTGGGAAACATCAAAGG	RT 42 °C 60 min; inactivation 70 °C 10 min; cooling 20 °C ∞
1st PCR	1a fw	1435–1454	TGGCTGGGAAACATCAAAGG	95 °C 1 min; (94 °C 20 s, 54 °C 20 s, 72 °C 45 s) × 40 cycles; 72 °C 3 min
	1a rv	308–326	CCTCCAGAGGACCCCTTCA	
M13 PCR	M13-fw	883–900	CACAGGAAACAGCTATGACCTCGGCATGGCATCTCCAC	95° C 2 min; (94 °C 20 s, 60 °C 20 s, 72 °C 30 s) × 35 cycles; 72 °C 3 min
	M13-rv	663–683	GTTGTAAAACGACGGCCAGTCGCGTTCCATCCTTTCTTACC	
MID PCR	MID fw	-	MID-GTTGTAAAACGACGGCCAGT	95 °C 2 min; (94 °C 20 s, 60 °C 20 s, 72 °C 45 s) × 25 cycles; 72 °C 3 min
	MID rv	-	MID-CACAGGAAACAGCTATGACC	

**Table 2:** Main clinical and viral characteristics of chronic hepatitis delta (CHD) patients included in the study. The table shows the clinical and viral characteristics of the patients between the two timepoints (Sample A and Sample B). P values were obtained by applying the Kruskal–Wallis test. Abbreviations (normal quantification values): HDV, Hepatitis delta virus, AST, Aspartate aminotransferase (normal value 12–50 IU/mL); ALT, alanine aminotransferase (normal value 8–50 IU/mL); platelets (140–400 UI/mL); IQR, interquartile range; HBsAg, hepatitis B virus surface antigen; IND, indetectable.

Markers	Sample A	Sample B	*p*
HDV RNA Median (IQR) Log10 (IU/mL)	5.76 (5.02–5.83)	5.76 (3.68–5.76)	0.383
AST (UI/L) Median (IQR)	88 (45–133)	90 (36.25–126)	0.551
ALT (UI/L) Median (IQR)	100 (58–158.5)	89.25 (44.5–133.75)	0.439
PLATELETS (UI/L) Median (IQR)	89 (123–212)	79 (118–197)	0.966
HBV-DNA (IU/mL)	Low/IND	Low/IND	
HBsAg Median (IQR) Log10 (IU/mL)	3.96 (3.57–4.11)	3.92 (3.39–4.03)	0.827

**Table 3:** Most prevalent mutations observed in the ribozyme. Table shows the 12 most prevalent mutations observed in the ribozyme. The relative frequency of each mutation between sample A and sample B is reported as mean ± standard deviation. Mutations are numbered starting from the first nt in the ribozyme (nt 688).

Mutation	Sample A (%freq)	Sample B (%freq)
G6A	0.84 ± 0.43%	0.83 ± 0.31%
T23C	3.04 ± 2.27%	5.55 ± 8.91%
T27C	0.12 ± 0.3%	0.88 ± 3.5%
G40A	0.25 ± 0.22%	0.41 ± 0.24%
G59A	0.07 ± 0.03%	0.08 ± 0.02%
T60C	6.15 ± 30.64%	6.42 ± 35.01%
C61T	0.15 ± 0.24%	0.14 ± 0.27%
C62T	0.12 ± 0.17%	0.08 ± 0.12%
C64d	47.17 ± 1.66%	48.88 ± 2.47%
T65C	6.02 ± 39.66%	6.29 ± 44.04%
T69C	12.02 ± 21.99%	12.75 ± 44.04%
G76A	0.53 ± 2.49%	0.44 ± 17.68%

## Data Availability

NGS data were submitted to the GenBank SRA database (BioProject accession number PRJNA798886). See Biosample numbers list in Supplementary Material.

## Supplementary Materials

The following are available online at https://www.mdpi.com/article/10.3390/v14020215/s1, Table S1: Protocols for delta antigen (HDAg) encoding HDV genome region amplification, Table S2: complexity indexes calculated in samples A and B in the ribozyme region, Figure S1: Number and type of mutations found in the ribozyme region, Figure S2: Evolution of mutation frequency between the two follow-up samples (A and B).

## References

[1] Sureau C., Negro F. (2016). The Hepatitis Delta Virus: Replication and Pathogenesis. J. Hepatol..

[2] Chen H.-Y., Shen D.-T., Ji D.-Z., Han P.-C., Zhang W.-M., Ma J.-F., Chen W.-S., Goyal H., Pan S., Xu H.-G. (2019). Prevalence and Burden of Hepatitis D Virus Infection in the Global Population: A Systematic Review and Meta-Analysis. Gut.

[3] Wedemeyer H., Negro F. (2019). Devil Hepatitis D: An Orphan Disease or Largely Underdiagnosed?. Gut.

[4] Wang T.-C., Chao M. (2003). Molecular Cloning and Expression of the Hepatitis Delta Virus Genotype IIb Genome. Biochem. Biophys. Res. Commun..

[5] Farci P., Anna Niro G. (2018). Current and Future Management of Chronic Hepatitis D. Gastroenterol. Hepatol..

[6] Sharmeen L., Kuo M.Y., Dinter-Gottlieb G., Taylor J. (1988). Antigenomic RNA of Human Hepatitis Delta Virus Can Undergo Self-Cleavage. J. Virol..

[7] Reid C.E., Lazinski D.W. (2000). A Host-Specific Function Is Required for Ligation of a Wide Variety of Ribozyme-Processed RNAs. Proc. Natl. Acad. Sci. USA.

[8] Wu H.N., Lin Y.J., Lin F.P., Makino S., Chang M.F., Lai M.M. (1989). Human Hepatitis Delta Virus RNA Subfragments Contain an Autocleavage Activity. Proc. Natl. Acad. Sci. USA.

[9] Sharmeen L., Kuo M.Y., Taylor J. (1989). Self-Ligating RNA Sequences on the Antigenome of Human Hepatitis Delta Virus. J. Virol..

[10] Kruger K., Grabowski P.J., Zaug A.J., Sands J., Gottschling D.E., Cech T.R. (1982). Self-Splicing RNA: Autoexcision and Autocyclization of the Ribosomal RNA Intervening Sequence of Tetrahymena. Cell.

[11] Golden B.L. (2011). Two Distinct Catalytic Strategies in the Hepatitis Delta Virus Ribozyme Cleavage Reaction. Biochemistry.

[12] Tang J., Breaker R.R. (2000). Structural Diversity of Self-Cleaving Ribozymes. Proc. Natl. Acad. Sci. USA.

[13] Ferré-D’Amaré A.R., Zhou K., Doudna J.A. (1998). Crystal Structure of a Hepatitis Delta Virus Ribozyme. Nature.

[14] Riccitelli N., Lupták A. (2013). HDV Family of Self-Cleaving Ribozymes. Prog. Mol. Biol. Transl. Sci..

[15] Thill G., Vasseur M., Tanner N.K. (1993). Structural and Sequence Elements Required for the Self-Cleaving Activity of the Hepatitis Delta Virus Ribozyme. Biochemistry.

[16] Been M.D., Wickham G.S. (1997). Self-Cleaving Ribozymes of Hepatitis Delta Virus RNA. Eur. J. Biochem..

[17] Quer J., Rodríguez-Frias F., Gregori J., Tabernero D., Soria M.E., García-Cehic D., Homs M., Bosch A., Pintó R.M., Esteban J.I. (2017). Deep Sequencing in the Management of Hepatitis Virus Infections. Virus Res..

[18] Domingo E., Perales C. (2019). Viral Quasispecies. PLoS Genet..

[19] Orito E., Mizokami M., Ina Y., Moriyama E.N., Kameshima N., Yamamoto M., Gojobori T. (1989). Host-Independent Evolution and a Genetic Classification of the Hepadnavirus Family Based on Nucleotide Sequences. Proc. Natl. Acad. Sci. USA.

[20] Revill P.A., Tu T., Netter H.J., Yuen L.K.W., Locarnini S.A., Littlejohn M. (2020). The Evolution and Clinical Impact of Hepatitis B Virus Genome Diversity. Nat. Rev. Gastroenterol. Hepatol..

[21] Tabernero D., Cortese M.F., Buti M., Rodriguez-Frias F. (2018). HDV Evolution—Will Viral Resistance Be an Issue in HDV Infection?. Curr. Opin. Virol..

[22] Homs M., Rodriguez-Frias F., Gregori J., Ruiz A., Reimundo P., Casillas R., Tabernero D., Godoy C., Barakat S., Quer J. (2016). Evidence of an Exponential Decay Pattern of the Hepatitis Delta Virus Evolution Rate and Fluctuations in Quasispecies Complexity in Long-Term Studies of Chronic Delta Infection. PLoS ONE.

[23] Lee C.-M., Bih F.-Y., Chao Y.-C., Govindarajan S., Lai M.M.C. (1992). Evolution of Hepatitis Delta Virus Rna during Chronic Infection. Virology.

[24] le Gal F., Gault E., Ripault M.-P., Serpaggi J., Trinchet J.-C., Gordien E., Deny P. (2006). Eighth Major Clade for Hepatitis Delta Virus. Emerg. Infect. Dis..

[25] Dény P. (2006). Hepatitis Delta Virus Genetic Variability: From Genotypes I, II, III to Eight Major Clades?. Hepatitis Delta Virus.

[26] Homs M., Caballero A., Gregori J., Tabernero D., Quer J., Nieto L., Esteban R., Buti M., Rodriguez-Frias F. (2014). Clinical Application of Estimating Hepatitis B Virus Quasispecies Complexity by Massive Sequencing: Correlation between Natural Evolution and On-Treatment Evolution. PLoS ONE.

[27] Sopena S., Godoy C., Tabernero D., Homs M., Gregori J., Riveiro-Barciela M., Ruiz A., Esteban R., Buti M., Rodríguez-Frías F. (2018). Quantitative Characterization of Hepatitis Delta Virus Genome Edition by Next-Generation Sequencing. Virus Res..

[28] Garcia-Garcia S., Cortese M.F., Rodríguez-Algarra F., Tabernero D., Rando-Segura A., Quer J., Buti M., Rodríguez-Frías F. (2021). Next-Generation Sequencing for the Diagnosis of Hepatitis B: Current Status and Future Prospects. Expert Rev. Mol. Diagn..

[29] Lempp F.A., Ni Y., Urban S. (2016). Hepatitis Delta Virus: Insights into a Peculiar Pathogen and Novel Treatment Options. Nat. Rev. Gastroenterol. Hepatol..

[30] Scheller L., Hilgard G., Anastasiou O., Dittmer U., Kahraman A., Wedemeyer H., Deterding K. (2021). Poor Clinical and Virological Outcome of Nucleos(t)Ide Analogue Monotherapy in HBV/HDV Co-Infected Patients. Medicine.

[31] Kang C., Syed Y.Y. (2020). Bulevirtide: First Approval. Drugs.

[32] Abbas Z. (2015). Management of Hepatitis Delta: Need for Novel Therapeutic Options. World J. Gastroenterol..

[33] Wang S.-Y., Wu J.-C., Chiang T.-Y., Huang Y.-H., Su C.-W., Sheen I.-J. (2007). Positive Selection of Hepatitis Delta Antigen in Chronic Hepatitis D Patients. J. Virol..

[34] Paul-Ehrlich-Institut A WHO Collaborating Centre Bundesinstitut Für Impfstoffe Und Biomedizinische Arzneimittel for Quality Assurance of Blood Products and Federal Institute for Vaccines and Biomedicines in Vitro Diagnostic Devices. https://www.pei.de/SharedDocs/Downloads/EN/regulation-en/referencematerial/7657-12-ifu.pdf?__blob=publicationFile&v=2.

[35] R Core Team (2020)—European Environment Agency. https://www.eea.europa.eu/data-and-maps/indicators/oxygen-consuming-substances-in-rivers/r-development-core-team-2006.

[36] Cortese M.F., González C., Gregori J., Casillas R., Carioti L., Guerrero-Murillo M., Riveiro-Barciela M., Godoy C., Sopena S., Yll M. (2021). Sophisticated Viral Quasispecies with a Genotype-Related Pattern of Mutations in the Hepatitis B X Gene of HBeAg-ve Chronically Infected Patients. Sci. Rep..

[37] Godoy C., Tabernero D., Sopena S., Gregori J., Francesca Cortese M., González C., Casillas R., Yll M., Rando A., López-Martínez R. (2019). Characterization of Hepatitis B Virus X Gene Quasispecies Complexity in Mono-Infection and Hepatitis Delta Virus Superinfection. World J. Gastroenterol..

[38] Wu T.T., Netter H.J., Lazinski D.W., Taylor J.M. (1997). Effects of Nucleotide Changes on the Ability of Hepatitis Delta Virus to Transcribe, Process, and Accumulate Unit-Length, Circular RNA. J. Virol..

[39] Homs M., Buti M., Quer J., Jardi R., Schaper M., Tabernero D., Ortega I., Sanchez A., Esteban R., Rodriguez-Frias F. (2011). Ultra-Deep Pyrosequencing Analysis of the Hepatitis B Virus PreCore Region and Main Catalytic Motif of the Viral Polymerase in the Same Viral Genome. Nucleic Acids Res..

[40] Yll M., Cortese M.F., Guerrero-Murillo M., Orriols G., Gregori J., Casillas R., González C., Sopena S., Godoy C., Vila M. (2020). Conservation and Variability of Hepatitis B Core at Different Chronic Hepatitis Stages. World J. Gastroenterol..

[41] Chang M.F., Chen C.H., Lin S.L., Chen C.J., Chang S.C. (1995). Functional Domains of Delta Antigens and Viral RNA Required for RNA Packaging of Hepatitis Delta Virus. J. Virol..

[42] Shirvani-Dastgerdi E., Amini-Bavil-Olyaee S., Alavian S.M., Trautwein C., Tacke F. (2015). Comprehensive Analysis of Mutations in the Hepatitis Delta Virus Genome Based on Full-Length Sequencing in a Nationwide Cohort Study and Evolutionary Pattern during Disease Progression. Clin. Microbiol. Infect..

[43] Mentha N., Clément S., Negro F., Alfaiate D. (2019). A Review on Hepatitis D: From Virology to New Therapies. J. Adv. Res..

[44] Loureiro D., Castelnau C., Tout I., Boyer N., Narguet S., Menasria Benazzouz S., Louis Z., Pons-Kerjean N., Giuly N., Marcellin P. (2021). New Therapies for Hepatitis Delta Virus Infection. Liver Int..

[45] Asselah T., Loureiro D., le Gal F., Narguet S., Brichler S., Bouton V., Abazid M., Boyer N., Giuly N., Gerber A. (2021). Early Virological Response in Six Patients with Hepatitis D Virus Infection and Compensated Cirrhosis Treated with Bulevirtide in Real-Life. Liver Int..

[46] Chang J., Taylor J.M. (2003). Susceptibility of Human Hepatitis Delta Virus RNAs to Small Interfering RNA Action. J. Virol..

[47] Li H., Mao Q., Li Q. (1999). Inhibitory Effect of Replication and Expression of HDV by Antisense Oligodeoxynucleotides in H1 Delta 9 Cell. Chin. J. Hepatol..

[48] Ye X., Tateno C., Thi E.P., Kakuni M., Snead N.M., Ishida Y., Barnard T.R., Sofia M.J., Shimada T., Lee A.C.H. (2019). Hepatitis B Virus Therapeutic Agent ARB-1740 Has Inhibitory Effect on Hepatitis Delta Virus in a New Dually-Infected Humanized Mouse Model. ACS Infect. Dis..

[49] Chad Y.-C., Chang M.-F., Gust I., Lai M.M.C. (1990). Sequence Conservation and Divergence of Hepatitis δ Virus RNA. Virology.

[50] Rosenstein S.P., Been M.D. (1996). Hepatitis Delta Virus Ribozymes Fold to Generate a Solvent-Inaccessible Core with Essential Nucleotides Near the Cleavage Site Phosphate. Biochemistry.

[51] Perrotta A.T., Been M.D. (1991). A Pseudoknot-like Structure Required for Efficient Self-Cleavage of Hepatitis Delta Virus RNA. Nature.

[52] Chadalavada D.M., Cerrone-Szakal A.L., Bevilacqua P.C. (2007). Wild-Type Is the Optimal Sequence of the HDV Ribozyme under Cotranscriptional Conditions. RNA.

[53] Perrotta A.T., Been M.D. (1996). Core Sequences and a Cleavage Site Wobble Pair Required for HDV Antigenomic Ribozyme Self-Cleavage. Nucleic Acids Res..

[54] Tanner N.K., Schaff S., Thill G., Petit-Koskas E., Crain-Denoyelle A.-M., Westhof E. (1994). A Three-Dimensional Model of Hepatitis Delta Virus Ribozyme Based on Biochemical and Mutational Analyses. Curr. Biol..

[55] Timm J., Lauer G.M., Kavanagh D.G., Sheridan I., Kim A.Y., Lucas M., Pillay T., Ouchi K., Reyor L.L., zur Wiesch J.S. (2004). CD8 Epitope Escape and Reversion in Acute HCV Infection. J. Exp. Med..

[56] Leslie A.J., Pfafferott K.J., Chetty P., Draenert R., Addo M.M., Feeney M., Tang Y., Holmes E.C., Allen T., Prado J.G. (2004). HIV Evolution: CTL Escape Mutation and Reversion after Transmission. Nat. Med..

[57] Cotrina M., Buti M., Jardi R., Quer J., Rodriguez F., Pascual C., Esteban R., Guardia J. (1998). Hepatitis Delta Genotypes in Chronic Delta Infection in the Northeast of Spain (Catalonia). J. Hepatol..

