# Supplementary material for: Inspecting the Ribozyme Region of Hepatitis Delta Virus Genotype 1: Conservation and Variability
Source: Viruses. 2022 Jan 22;14(2):215. doi: 10.3390/v14020215 (PMC8877431; doi:10.3390/v14020215)
Supplement: Supplementary file 1 [file viruses-14-00215-s001.zip › viruses-1547090-supplementary.pdf]

Supplementary Table S1. Protocols for delta antigen (HDAg) encoding HDV genome region amplification. *The table shows the different steps for amplification of the region of HDV genome encoding the HDAg used for HDV genotyping, including the retro-transcription and the three nested-PCR. Primer's sequence and amplification region per each step is reported. M13 sequence tail is underlined. Fw: Forward; Rv: Reverse; MID: multiplex identifier.*

Amplification step	Primer	Amplified region	Primer sequence (5′→3′)	Protocol
RT	RT rv	728-747	CGGTCCCCTCGGAATGTTG	RT 42 ºC 60 min; inactivation 70ºC 10 min; cooling 20ºC ∞
1st PCR	1a fw	865-884	AGGTCGGACCGCGAGGAGGT	95 ºC 1 min; (94 ºC 20 s. 54 ºC 20 s. 72 ºC 45s) × 40 cycles; 72 ºC 3 min
	1a rv	306-328	GCTGAAGGGGTCCTCTGGAGGTG	
M13 PCR	M13 fw	886-909	GTTGTAAAACGACGGCCAGTGAGATGCCATGCCGACCCGAAGAG	95 ºC 2 min; (94 ºC 20 s. 63 ºC 20 s. 72 ºC 30s) × 35 cycles; 72 ºC 3 min
	M13 rv	1272-1295	CACAGGAAACAGCTATGACCCGACGAAGGAAGGCCCTCGAGAAC	
MID PCR	MID fw	-	MID-GTTGTAAAACGACGGCCAGT	95 ºC 2 min; (94 ºC 20 s. 60 ºC 20 s. 72 ºC 45s) × 25 cycles; 72 ºC 3 min
	MID rv	-	MID-CACAGGAAACAGCTATGACC	

Supplementary Table S2. Median complexity indexes calculated in samples A and B in the ribozyme region. *The table shows the median and IQR of the following indexes: number of reads per sample; number of master sequence reads (Mstr); percentage of the master sequence (Mpct); number of haplotypes; number of polymorphic sites; Shannon index; Gini-Simpson coefficient; functional attribute diversity (FAD); mutation frequency (Mf); nucleotide diversity (Pi) and Pi to Mf ratio. P-values were obtained by applying a Kruskal Wallis-test.*

Complexity index	Sample A Median (IQR)	Sample B Median (IQR)	*p*
**N reads**	3785 (1196.5 – 4981.5)	4909.5 (1550.5 – 6460)	.273
**N reads of master (Mstr)**	2462.5 (1109 – 3571.5)	3161.5 (1440 – 4601.5)	.389
**% master (Mpct)**	9.15 (85.17 – 94.33)	10.52 (83.9 – 94.4)	.872
**N haplotypes**	2.5 (6.5 – 9)	2.5 (6.5 – 9)	.737
**Polymorphic sites**	3 (5 – 8)	3 (5 – 8)	.907
**N mutations**	3 (5 – 8)	3 (5 – 8)	.895
**Shannon index**	0.32 (0.31 – 0.63)	0.41 (0.29 – 0.71)	.941
**Gini Simpson coefficient**	0.16 (0.1 – 0.26)	0.18 (0.1 – 0.28)	.872
**Functional attribute diversity (FAD)**	0.89 (0.7 -1.6)	0.77 (0.1 – 1.48)	.569
**Mutation frequency (Mf)**	0.001 (0.0006 – 0.0017)	0.0012 (0.0006 – 0.0018)	.872
**Nucleotide diversity (Pi)**	0.002 (0.0012 – 0.0033)	0.0023 (0.0012 – 0.0036)	.918
**Pi to Mf ratio**	0.10 (1.86 – 1.97)	0.11 (1.86 – 1.97)	.815

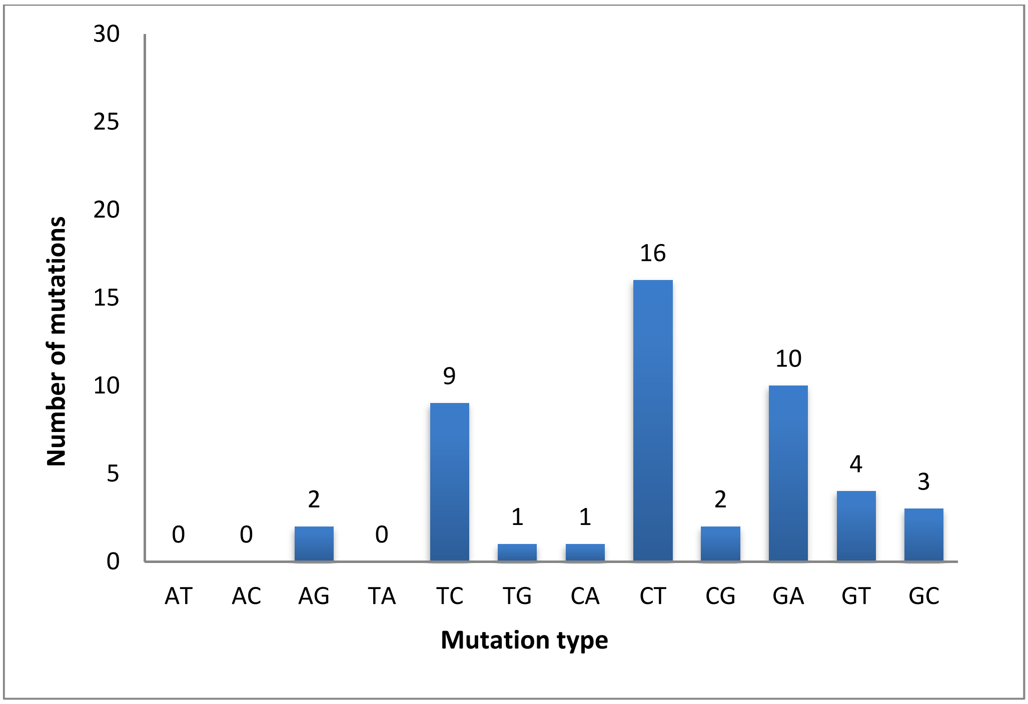

***Supplementary Figure S1. Number and type of mutations found in the ribozyme region**. The barplot shows the total number of each type of nucleotide change within the sequence.*

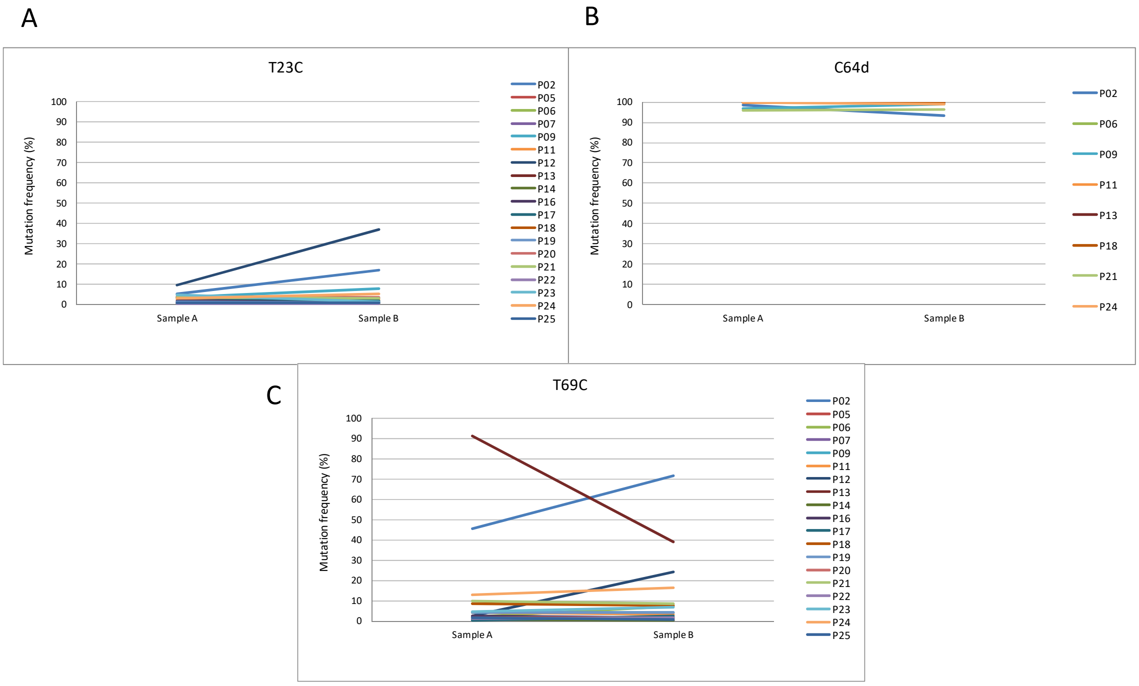

***Supplementary Figure S2. Evolution of mutation frequency between the two follow-up samples (A and B)**. Each patient is represented by a different color. The x-axis represents the two samples of each patient from the follow-up; the basal (Sample A) and the follow-up (Sample B) and the y-axis shows the relative frequency (%) in which the mutation pattern is present in each sample of each patient. T23C mutation; Panel A, C64 deletion; Panel B and T69C mutation; Panel C.*
